# Peptide extract from spent yeast improves resistance of *Saccharomyces cerevisiae* to oxidative stress

**DOI:** 10.1007/s00253-023-12514-3

**Published:** 2023-04-22

**Authors:** Ana Lopes, João Azevedo-Silva, Erdem Carsanba, Manuela Pintado, Ana Sofia Oliveira, Carlos Ferreira, Joana Odila Pereira, Ana P. Carvalho, Carla Oliveira

**Affiliations:** 1 Amyris BioProducts Portugal, Unipessoal, Lda. Rua Diogo Botelho 1327, 4169-005 Porto, Portugal; 2 Universidade Católica Portuguesa, CBQF - Centro de Biotecnologia e Química Fina – Laboratório Associado, Escola Superior de Biotecnologia, Rua Diogo Botelho 1327, 4169-005 Porto, Portugal

**Keywords:** Antioxidant activity, Bioreactor fermentation, Reactive oxygen species, Farnesene production, Peptide-rich extract, Circular economy

## Abstract

**Abstract:**

Yeast cells face various stress factors during industrial fermentations, since they are exposed to harsh environmental conditions, which may impair biomolecules productivity and yield. In this work, the use of an antioxidant peptide extract obtained from industrial spent yeast was explored as supplement for *Saccharomyces cerevisiae* fermentation to prevent a common bottleneck: oxidative stress. For that, a recombinant yeast strain, producer of β-farnesene, was firstly incubated with 0.5 and 0.7 g/L peptide extract, in the presence and absence of hydrogen peroxide (an oxidative stress inducer), for 1–5 h, and then assayed for intracellular reactive oxygen species, and growth ability in agar spot assays. Results showed that under 2 mM H_2_O_2,_ the peptide extract could improve cells growth and reduce reactive oxygen species production. Therefore, this antioxidant effect was further evaluated in shake-flasks and 2-L bioreactor batch fermentations. Peptide extract (0.7 g/L) was able to increase yeast resistance to the oxidative stress promoted by 2 mM H_2_O_2_, by reducing reactive oxygen species levels between 1.2- and 1.7-fold in bioreactor and between 1.2- and 3-fold in shake-flask fermentations. Moreover, improvements on yeast cell density of up to 1.5-fold and 2-fold, and on biomolecule concentration of up to 1.6-fold and 2.8-fold, in bioreactor and shake-flasks, respectively, were obtained. Thus, culture medium supplementation with antioxidant peptide extracted from industrial spent yeast is a promising strategy to improve fermentation performance while valuing biomass waste. This valorization can promote a sustainable and eco-friendly solution for the biotechnology industry by the implementation of a circular economy model.

**Key points:**

*• Peptide extract from spent yeast applied for the first time on yeast fermentation.*

*• Antioxidant peptide extract enhanced S. cerevisiae oxidative stress resistance.*

*• Fermentation performance under stress improved by peptide extract supplementation.*

## Introduction

Industrial biotechnology sights the production of chemicals, proteins, metabolites, biofuels, and other products in a sustainable manner. *Saccharomyces cerevisiae* is widely used and studied in industrial fermentation due its fast growth rate, strong ability to adapt to large scale fermentation, high tolerance against harsh conditions, and relatively easy genetic manipulation (Carsanba et al. 2021; Nandy and Srivastava 2018; Stalidzans and Dace 2021). Microbial fermentation allows the production of natural molecules with high yield, low environmental impact, and without exploring natural resources (Nandy and Srivastava 2018). Despite these numerous advantages, industrial fermentation also produce waste. One of the major wastes is spent yeast biomass, which also contains compounds with commercial interest, due to their several biological activities (antihypertensive, antioxidant, and antimicrobial activities) (Oliveira et al. 2022a; San Martin et al. 2021). Thus, spent yeast is a valuable fermentation residue for extracting biologically active compounds for distinct applications, including dietary supplements and cosmetics (Oliveira et al. 2022a).

During industrial fermentations, yeast cells face several environmental stresses such as heat, osmotic, oxidative, pH, and ethanol, that can impair their fermentation efficiency (Deparis et al. 2017; Saini et al. 2018). Some of these harsh conditions can trigger the accumulation of intracellular reactive oxygen species (ROS), which promote yeast oxidative stress (Saini et al. 2018; Walker and Basso 2020). Nevertheless, ROS are free radicals generated also by intracellular metabolism processes, like aerobic respiration in mitochondria. Oxidative stress can lead to protein oxidation, lipid peroxidation, DNA damage, and thus triggering programmed cell death (apoptosis) (Deparis et al. 2017; Saini et al. 2018).

Under normal physiological conditions, ROS are neutralized by the activation of enzymatic and non-enzymatic defence mechanisms to maintain the cellular redox balance and to resist oxidative stress (Auesukaree 2017; Deparis et al. 2017; Oliveira et al. 2022a). The antioxidant enzymatic defence system consists of a set of protective enzymes (superoxide dismutases (SODs), catalases (CATs), glutathione peroxidases (GPXs), and peroxiredoxins (Prxs)) which can be upregulated to detoxify ROS and maintain the intracellular redox environment in a reduced state (Auesukaree 2017; Deparis et al. 2017; Eigenfeld et al. 2021). These enzymes develop a complex and tight detoxification process that starts when the SODs convert superoxide anion to hydrogen peroxide (H_2_O_2_), which is then reduced to water and oxygen (Auesukaree 2017; San Martin et al. 2021). The non-enzymatic defence mechanisms correspond to small molecules, such as glutathione, D-erythroascorbate, trehalose, and ubiquinol, which act as radical scavengers (Auesukaree 2017).

However, when ROS levels are too high, the endogenous defence mechanisms are not enough to protect the cells from oxidative damages. The most described methods to prevent oxidative damage are medium optimization and yeast engineering. The first one is through the addition of known antioxidants, such as phenolic and carotenoid compounds into culture medium (Carvalho et al. 2022; Mendes et al. 2015; Qiu et al. 2019). The supplementation of medium with antioxidant divalent metal ions (such as Zn^2+^, Ca^2+^, and Mg^2+^) and amino acids (such as arginine, proline, tyrosine, and tryptophan) is also a common strategy to improve yeast stress tolerance (Qiu et al. 2019; Takagi 2021). On the other hand, the overexpression of proteins and enzymes with antioxidant capacity, like CAT, SOD, and GSH, or deleting genes such as proline dehydrogenase (Put1) can also lead to reduced cell stress (Li et al. 2020; Qiu et al. 2019).

A possible and unexplored application of the antioxidant compounds extracted from spent yeast would be their use in fermentation as an antioxidant supplement to reduce yeast oxidative stress. Reusing spent yeast to extract bioactive compounds allows the valorization of fermentation waste and promotes a circular economy policy (Faustino et al. 2021). Thus, the application of these extracts in yeast fermentation may improve the profitability of the fermentation process, by both bio-valorizing fermentation waste and improving yeast performance in a circular economy approach. According to that, this work aims to study the capacity of an antioxidant peptide-rich extract (APE), isolated from Amyris Inc., Emeryville, CA, USA, spent yeast (Oliveira et al. 2022b), to reduce oxidative stress in *S. cerevisiae* fermentation. In order to accomplish that, a genetically modified yeast producer of β-farnesene was exposed to 2 mM H_2_O_2_ (a stress inducer), in the presence and absence of APE, during shake-flasks and bioreactor fermentations. β-farnesene is currently produced by yeast fermentation at industrial scale (200,000 m^3^) (Benjamin et al. 2016; Meadows et al. 2016), in which the yeast faces elevated oxidative stress. As such, a recombinant yeast producer of β-farnesene, precursor of the yeast strains that have been used for farnesene manufacturing in recent years by Amyris Inc., was chosen as a model for this work. Besides the variations on the intracellular ROS levels, the antioxidant effect of APE on yeast growth and biomolecule production was also evaluated.

## Materials and methods

### Strain and culture media

The strain used in this study was a *S. cerevisiae* (strain: CEN.PK2, BioSample: SAMN27162618) engineered by Amyris Inc., Emeryville, USA, to produce β-farnesene constitutively. A pre-culture of this strain was grown in 63 mL of culture medium in one of the 250-mL Erlenmeyer flasks (with four baffles), inoculated with two cryovial (containing 1 mL of culture volume each), and incubated at 30 °C and 200 rpm in an orbital incubator for 42 h. The standard medium culture contained yeast extract (5 g/L), succinic acid buffer (6 g/L), potassium phosphate monobasic (1 g/L), magnesium sulfate heptahydrate (0.5 g/L), sucrose (60 g/L), and trace metals and vitamins as described by Hoek et al. (2000). Agar plates contained tryptophan (0.02 g/L), yeast nitrogen base without amino acids (6.7 g/L), yeast synthetic drop-out medium supplements (2 g/L), sucrose (2 g/L), maltose (1 g/L), and lysine (2 g/L). All reagents used in this study were from Sigma Aldrich, (St. Louis, MO, USA). APE was produced from spent yeast waste, resulting of Amyris Inc., Emeryville, USA, fermentations, by selective membrane filtration (Oliveira et al. 2022b).

### APE extraction

Peptide-rich extracts were obtained from waste streams of mannan (Mpep) extraction from spent yeast (*S. cerevisiae*) as described in the paper by Oliveira et al. (2022b). APE corresponded to the Mpep > 1 kDa and contained 86.4 ± 8.7% (w/w) protein, 7.44 ± 0.22% (w/w) of sugars and minerals (5.22 ± 0.01 ng/g extract of potassium, 1.04 ± 0.01 ng/g extract of magnesium, and 0.10 ± 0.01 ng/g extract of calcium).

### Antioxidant activity

Antioxidant activity of APE was determined by 2,2′-azinobis(3-ethyl-benzothiazoline-6-sulphonate)-radical (ABTS +) scavenging activity as described by Oliveira et al. (2022b).

### Spot assay

First, an optimization of oxidative stress induction in *S. cerevisiae* was performed for different H_2_O_2_ concentrations (1, 2, 5, and 10 mM), and times of exposure, according to the literature (Khan et al. 2005; Pedreño et al. 2002; Coelho et al. 2019). The following experiments were conducted using 2 and 5 mM H_2_O_2_ (with one and 5 h of incubation) since these were the conditions presenting the best impact on yeast membrane integrity and ROS production. Furthermore, the concentrations of APE selected were based on its water solubility, which was ≤ 0.7 g/L.

**Fig. 1:**
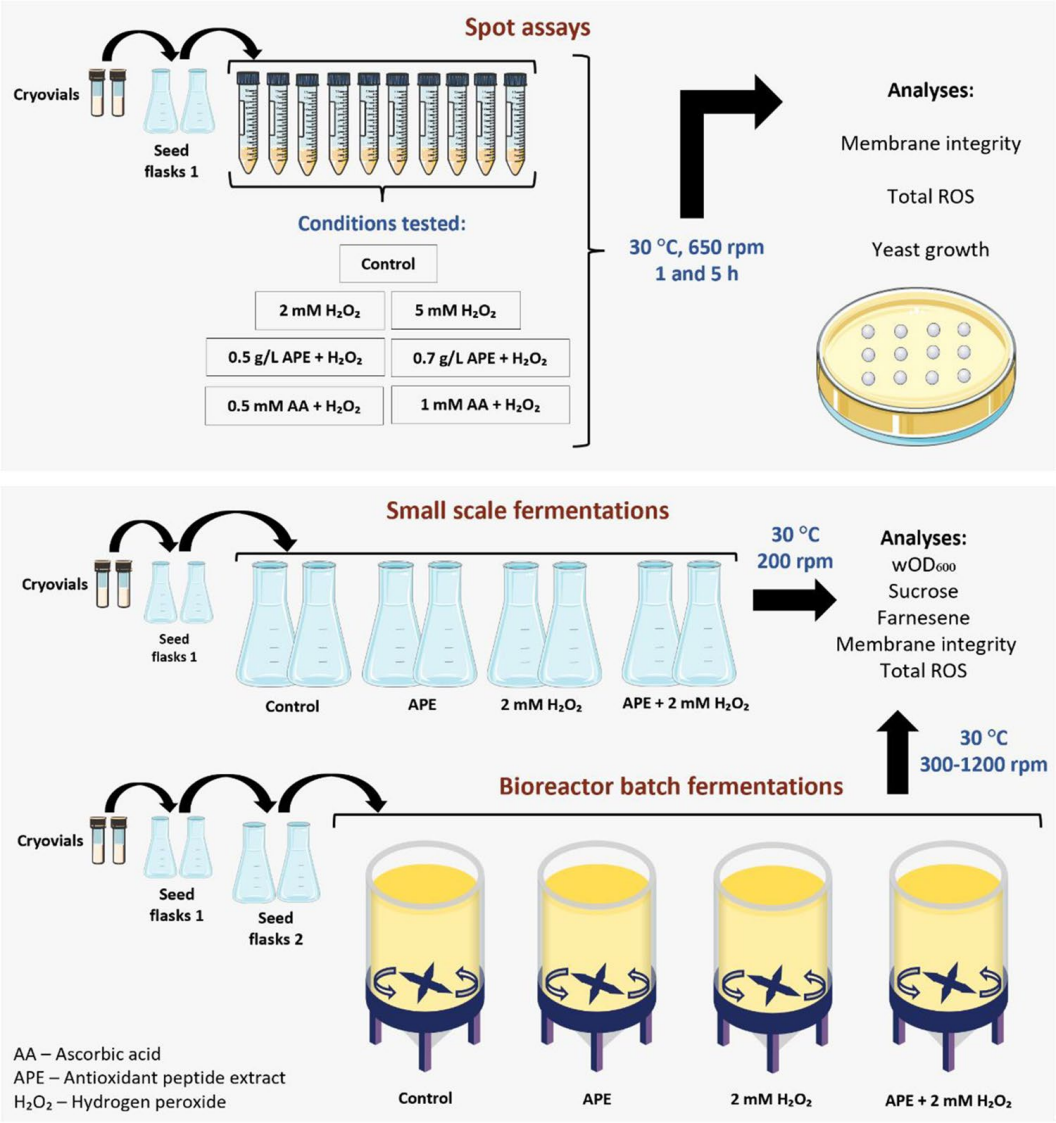
Schematic representation of spot assays after 2 and 5 h of exposure to hydrogen peroxide (H_2_O_2_) in the presence and absence of ascorbic acid (AA) or antioxidant peptide extract (APE) in 15 mL Falcons, small-scale fermentations (in 1 L shake-flasks) and bioreactor fermentations (in bioreactors with 2.7 L of working volume)

Pre-culture was harvested and suspended in 3 mL of liquid culture medium in 15 mL Falcons to an initial washed optical density at 600 nm (wOD_600_) of 1.0. In the control Falcon, cells were cultivated using standard culture medium, while in the other Falcons, cells were suspended in medium containing 2 mM or 5 mM H_2_O_2_ with and without the addition of 0.5 or 0.7 g/L APE and in medium containing 2 mM or 5 mM H_2_O_2_ with and without the addition of 0.5 and 1 mM of ascorbic acid (AA) (Fig. 1). Prepared Falcons were incubated for 5 h at 30 °C and 650 rpm on a thermomixer.

After 1 and 5 h of H_2_O_2_ exposure, cells were harvested by centrifugation at 13,500 rpm for 5 min, diluted in sterile 0.9% NaCl to wOD_600_ of 1.0, and then serially tenfold diluted. One aliquot (4 μL) of each dilution from each sample was transferred to agar plates and incubated at 30 °C for 4 days.

### Small scale (shake-flask) fermentation

Pre-culture cells were harvested and suspended in 100 mL of culture medium in several 1 L Erlenmeyer flasks (with 4 baffles) to an initial wOD_600_ of 0.5. The conditions tested in shake-flasks were standard culture medium (control), medium with 2 mM H_2_O_2_, medium with 0.7 g/L of APE (higher concentration possible due to water solubility), and medium with 0.7 g/L of APE plus 2 mM H_2_O_2_ (Fig. 1). APE was dissolved in deionized water, mixed with culture medium, and sterilized by filtration. Shake-flasks were incubated in an orbital incubator at 30 °C and 200 rpm until the sugars in medium were consumed. Samples were collected in each 3 h of a working day to determine washed optical density, farnesene concentration, and sugar consumption. Membrane integrity and ROS levels were evaluated in the first sample of each day. Two independent replicates were performed for shake-flasks fermentations. The execution of a third replicate was replaced by the scale-up to bioreactor fermentations to validate the positive effect of APE at a larger scale.

### Bioreactor batch fermentation

In order to obtain the inoculum for the batch bioreactor fermentations, two seed flask passages (incubation at 30 °C and 200 rpm) were performed. Incubation times in the first and second seed flask steps were 42 h and 22 h, respectively.

The batch fermentations were performed in reactors (Eppendorf DASGIP parallel bioreactor system, Hamburg, Germany) with 2.7 L of working volume maintaining temperature at 30 °C, pH at 5.0 (adjusted with ammonium hydroxide at 12.5%), aeration at 0.5 L/min, and dissolved oxygen (DO) of at least 30% (controlled by agitation ranging between 300 and 1200 rpm). Batch reactors had an initial volume of 1-L after inoculation (10% inoculum, initial wOD_600_ of ~ 1). Cells were cultured until all sugars in medium were consumed (~ 47 h). Different conditions were tested: reactor 1 contained the standard medium (control), reactor 2 medium with 2 mM H_2_O_2_, reactor 3 medium with 0.7 g/L APE, and reactor 4 medium with 0.7 g/L APE plus 2 mM H_2_O_2_ (Fig. 1).

Temperature (°C), pH, DO, agitation, aeration, and off-gas data, such as CO_2_, O_2_, oxygen uptake rate (OUR) and ethanol, were monitored and recorded automatically. Whole cell broth samples were collected and prepared for the measurements of farnesene concentration (g/kg), washed optical density, sucrose (g/L), ROS, and cell viability (%).

### Washed optical density

Yeast growth was analyzed by the measurement of the wOD_600_ at the defined sampling points. Samples were centrifuged at 13,500 rpm for 5 min (1 mL), and after discarding the supernatant, the cells were suspended in 1 mL of deionized water and diluted to the absorbance read on the spectrophotometer (Shimadzu UV-1900 UV–VIS Spectrophotometer, Kyoto, Japan) at 600 nm.

### Sucrose quantification (g/L)

Sucrose concentrations were determined by high-performance liquid chromatography (HPLC). The supernatant collected during wOD analysis was diluted fivefold in deionized water, filtered, and analyzed in HPLC using the Aminex HPX-87H column (300 × 7.8 mm) with a pre-column (30 × 4.6 mm) and a refractive index detector (RID) (BioRad, Hercules, CA, USA). The column and detector temperatures were set at 50 °C and 35 °C, respectively. Mobile phase of 5 mM of H_2_SO_4_ was eluted at 0.6 mL min^−1^. Six standards with 0.3, 0.6, 1.25, 2.5, 5, and 10 g/L of sucrose were used for the calibration curve determination. Data acquisition was performed using Agilent OpenLab CDS A.01.12.165 (Agilent, Santa Clara, CA, USA).

### Membrane integrity

Cellular membrane integrity was determined using the fluorescent probe propidium iodide (IP, Sigma-Aldrich, St. Louis, MO, USA, reference P4864). Cells were incubated with 10 µM propidium iodide at 30 °C for 20 min, and then harvested and suspended in 0.9% NaCl (w/v). The cells used as positive controls were treated with 70% ethanol for 10 min prior to incubation with IP. Fluorescence intensity was measured by flow cytometry (BD Accuri™ C6 Plus Personal Flow Cytometer; Accuri, Ann Arbor, MI, USA) using the FL-2 channel (585/40 nm) and reading 20 000 events.

### Measurement of ROS

The oxidant-sensitive fluorescent probe dihydrorhodamine (DHR; Sigma-Aldrich, St. Louis, MO, USA) was used to determine the intracellular ROS levels. Cells were treated with 50 µM DHR in culture media for 1 h, and then washed with 0.9% NaCl twice and suspended in 200 µL of 0.9% NaCl. Cells treated with 20 µM carbonyl cyanide 3-chlorophenylhydrazone (CCCP) during 10 min before incubation with DHR were used as positive control. Fluorescence was measured in FL-1 channel (533/530 nm) in a BD Accuri™ C6 plus Personal Flow Cytometer. Data was acquired from a total of 20,000 events/samples from 2 independent assays. Quantification of intracellular ROS was expressed by relative fluorescence units (RFU) calculated in relation to sample control (unlabeled cells).

### Farnesene determination

Farnesene determination was performed by gas chromatography (GC), using an Agilent 8890 GC System with a flame ionization detector (FID) (Agilent, Santa Clara, CA, USA. Two different standard farnesene solutions (low and high concentrations) and the samples were injected after performing a farnesene extraction protocol. Briefly, farnesene extraction was applied by mixing fermentation broth with a solution containing 10% 2-butoxyethanol, 0.25% tetradecane, and 90% methanol at a proportion of 1:20 or 1:40 (shake-flask or reactor samples, respectively). Samples were injected in a separation column (Phenomenex ZB-5HT Inferno; Phenomenex, Torrance, CA, USA, with 30 + 5 m guardian, 0.25 mm and 0.25 µm film) with the column temperature ranging from 60 to 325 °C. Hydrogen was used as sample carrier gas. Data acquisition was performed using Agilent OpenLab CDS–Build 2.205.0.1344 (Agilent, Santa Clara, CA, USA.

### Statistical analysis

Normality of data was determined by Shapiro-Wilk test. Statistical comparisons were performed by two-way ANOVA, followed by the Dunnett’s multiple comparisons test for all flow cytometry analyses. APE results were compared to control results, while APE + H_2_O_2_ results were compared to H_2_O_2_ data. Concerning the number of replicates, both the process and the analytical examinations were performed in duplicate. All statistical calculations were performed with the GraphPad Prism version 8.0 for Windows (GraphPad Software, San Diego, CA, USA). In all cases, *p* values lower than 0.05 were considered significant.

## Results

### Antioxidant extract restores yeast growth in the presence of hydrogen peroxide

**Fig. 2:**
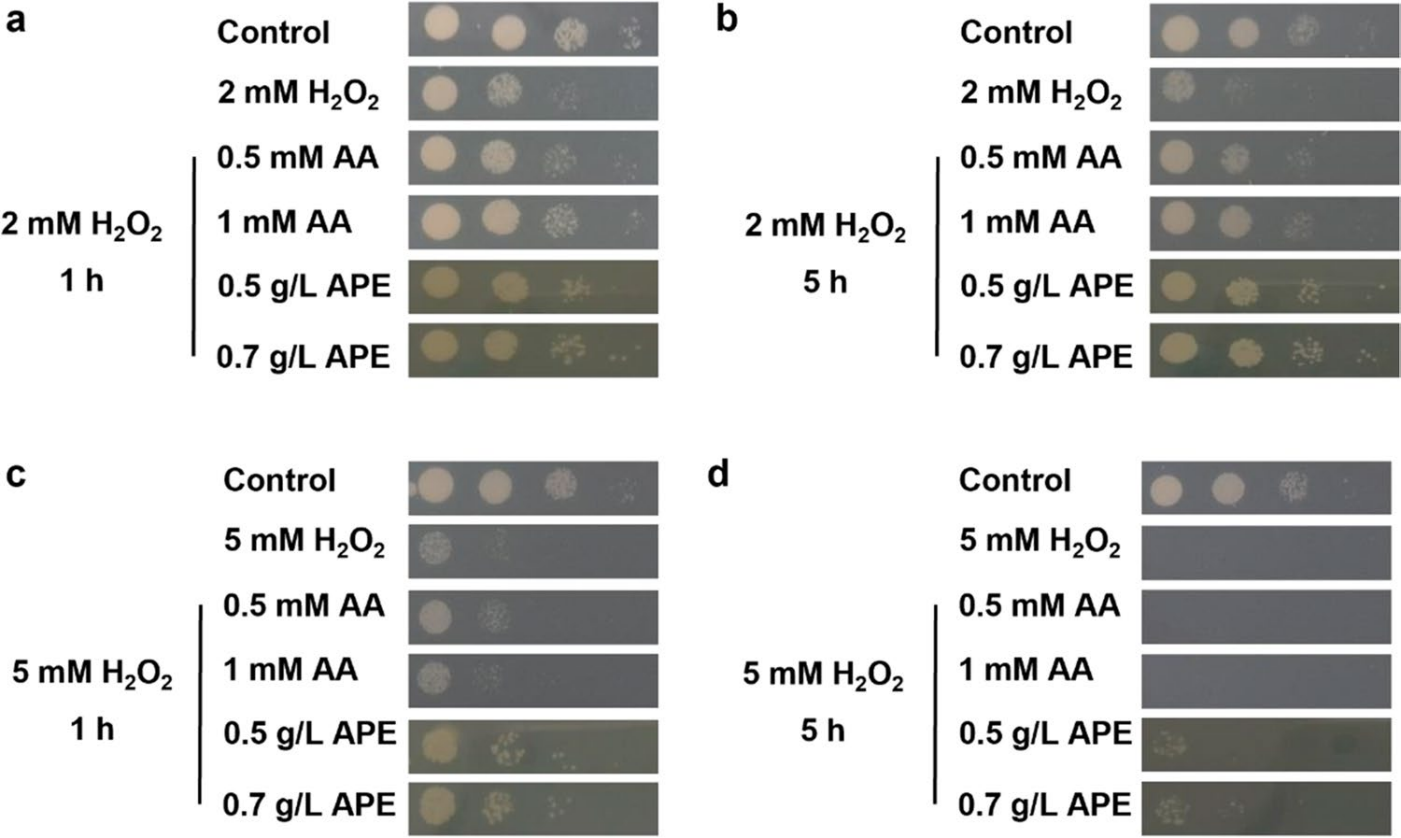
Yeast growth in the presence and absence of ascorbic acid (AA) or antioxidant peptide extract (APE) under hydrogen peroxide (H_2_O_2_) stress in agar plates, after 4 days of incubation at 30 °C. Cells were previously exposed to 2 mM H_2_O_2_ plus 0.5 mM AA, 1 mM AA, 0.5 g/L APE, and 0.7 g/L APE during 1 and 5 h (**a** and **b**, respectively). Cells were previously exposed to 5 mM H_2_O_2_ plus 0.5 mM AA, 1 mM AA, 0.5 g/L APE, and 0.7 g/L APE during 1 and 5 h (c and d). Three tenfold serial dilutions were done from an initial wOD = 1, and 4 µl of each dilution were spotted onto agar plates (wOD = 1, 10^−1^, 10^−2^, and 10.^−3^)

The supplementation of the culture medium with antioxidant compounds is a strategy to overcome oxidative stress during fermentation (Burphan et al. 2018; Mendes et al. 2015; Qiu et al. 2019). The capacity of an antioxidant peptide-rich extract produced from Amyris Inc., Emeryville, USA, spent yeast (Oliveira et al. 2022b) to reduce oxidative stress in *S. cerevisiae* during 1 and 5 h of H_2_O_2_ exposure was studied. Hydrogen peroxide, H_2_O_2_ (2 and 5 mM), was used as stress inducer to increase ROS generation. AA was used as a positive control since it is an antioxidant known to reduce oxidative stress in yeast (Eigenfeld et al. 2021; Takagi 2021). Yeast growth was evaluated in drops spotted in agar plates after 1 and 5 h of incubation at 30 °C in liquid medium containing 2 or 5 mM H_2_O_2_ in the presence of 0.5 and 1 mM AA, and 0.5 g/L and 0.7 g/L APE (Fig. 2).

Yeast growth was improved by the addition of 0.5 and 1 mM AA, as well by 0.5 and 0.7 g/L APE after 1 and 5 h of exposure to 2 mM H_2_O_2_ (Fig. 2a and b, respectively). On the other hand, after 1 and 5 h of exposure to 5 mM H_2_O_2_, APE (0.5 and 0.7 g/L) led to a slight improvement in yeast growth (Fig. 2c and d). According to drops spotted in agar plates, under stress conditions, 0.5 and 0.7 g/L APE enhanced yeast growth, possibly by aiding cells to overcome oxidative stress through ROS reduction during incubation. To confirm this hypothesis, intracellular ROS and membrane integrity under H_2_O_2_ stress in the presence and absence of APE were analyzed.

### Antioxidant compounds protect membrane integrity and decrease the level of intracellular ROS

**Fig. 3:**
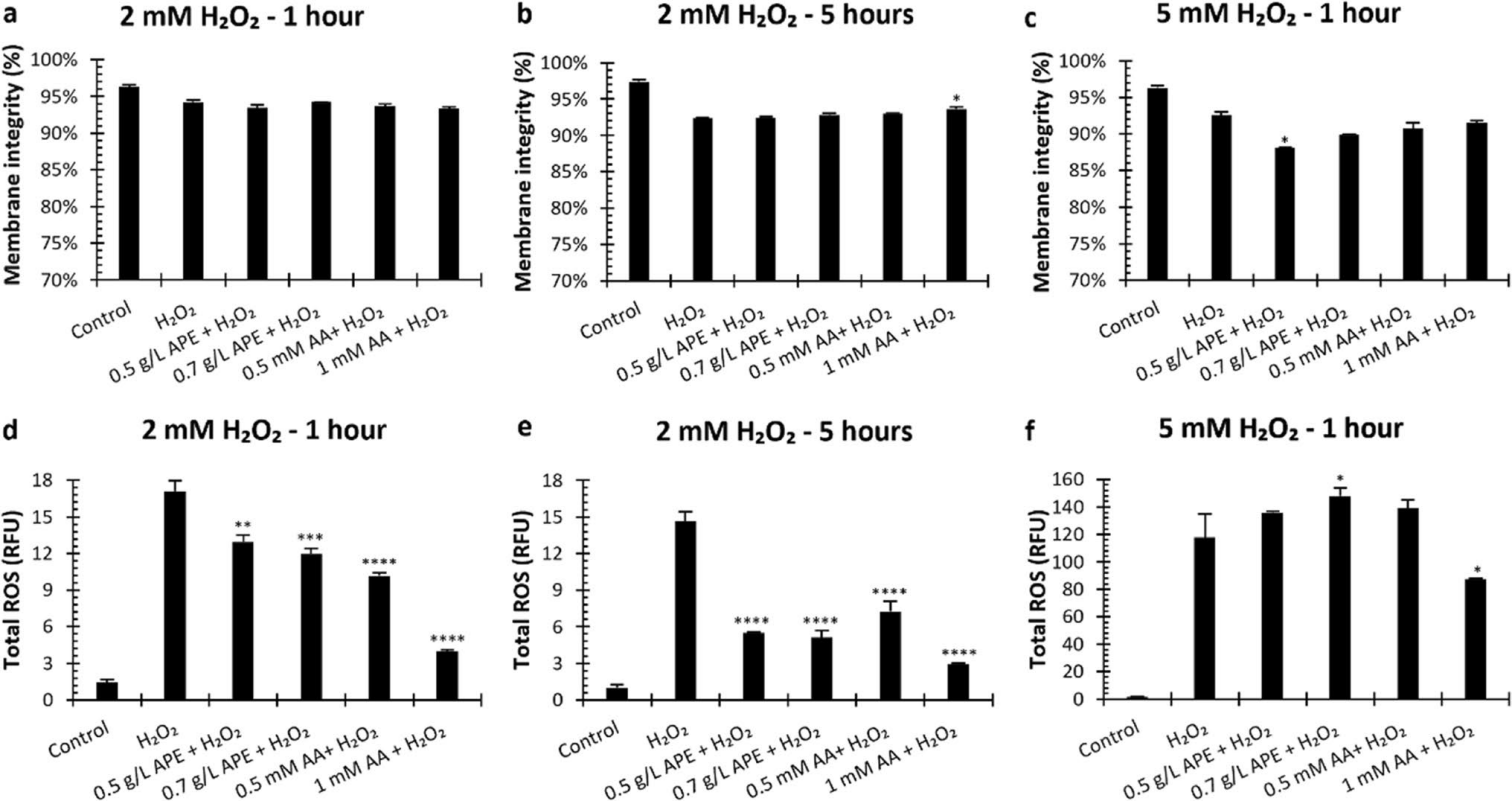
Membrane integrity and ROS production in yeast under hydrogen peroxide (H_2_O_2_) stress in presence of acetic acid (AA) or antioxidant peptide extract (APE). Membrane integrity was evaluated after 1 and 5 h of incubation with 2 mM H_2_O_2_ and 1 h with 5 mM H_2_O_2_ (**a**–**c**, respectively). Total ROS was also measured in fluorescence units relative to control (RFU) (**d**–**f**). The data represents the mean of two measurements for each condition, and error bars indicate standard deviations from the mean. Statistically significant differences were analyzed in all points using two-way ANOVA, followed by Dunnett’s multiple comparisons test (****p < 0.0001; ***p < 0.001; **p < 0.01; *p < 0.05 vs H_2_O_2_)

Membrane integrity and ROS levels were measured by flow cytometry, after 1 and 5 h of H_2_O_2_ exposure in the different conditions tested (Fig. 3).

Under H_2_O_2_ stress, membrane integrity was similar in the presence of antioxidant compounds, except in the condition 1 mM AA plus 2 mM H_2_O_2_, in which a significant high membrane integrity was observed after 5 h of exposure (Fig. 3a–c). On the other hand, under 2 mM H_2_O_2_ stress, supplementation of culture medium with APE and AA significantly improved yeast tolerance to oxidative stress (Fig. 3d and e). The addition of 0.5 g/L and 0.7 g/L of APE significantly decreased ROS levels by about 1.3 and 1.4-folds (17.08 RFU to 12.94 RFU and 11.97 RFU) after 1 h of incubation and 2.65 and 2.87-folds (14.62 RFU to 5.52 RFU and 5.1 RFU) after 5 h of exposure to H_2_O_2_, respectively. Thus, the higher reductions in ROS levels were promoted by 0.7 g/L APE. Moreover, 0.5 mM and 1 mM AA reduced ROS accumulation up to 1.68-fold (from 17.08 to 10.17 RFU) and 3.96-fold (from 17.08 to 4.31 RFU), respectively, after 1 h of 2 mM H_2_O_2_ exposure. After 5 h of incubation, in the presence of 2 mM H_2_O_2_, AA at 1 mM reduced ROS production from 14.62 to 6.24 RFU (2.0 times) and from 17.08 to 4.31 RFU (i.e., 4.92 times). Under 5 mM H_2_O_2_, ROS generation was high (117.62 RFU) and only 1 mM AA was capable to reduce ROS levels, to 87.16 RFU (Fig. 3f). Thus, in the presence of 2 mM H_2_O_2_, APE addition resulted in a significant reduction of ROS generated, which can be explained by its reported antioxidant properties (Oliveira et al. 2022b).

### Impact of APE in small-scale fermentation

**Fig. 4:**
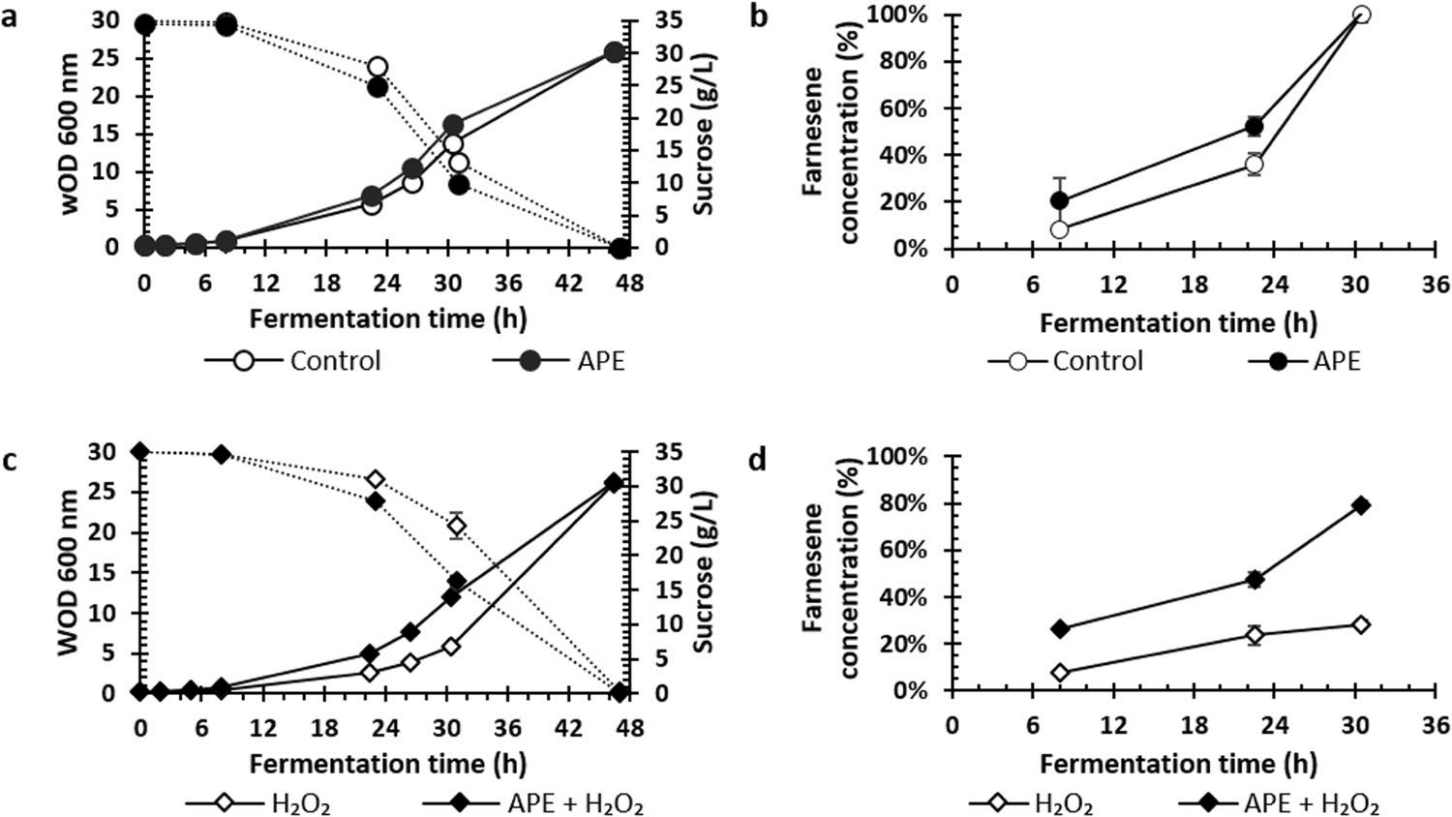
Growth curve (washed optical density at 600 nm — wOD_600_) and sucrose (g/L), round dot dash, during 47 h of shake-flask fermentation in the absence (**a**) and presence (**c**) of stress factor (hydrogen peroxide — H_2_O_2_) and antioxidant peptide extract (APE). Farnesene (%) (**b** and **d**). Farnesene results of Fig. 4b and d were both normalized for the control result of Fig. 4b at 30.5 h, to demonstrate the effect of oxidative stress on farnesene production relatively to standard condition used in farnesene fermentations. Furthermore, Fig. 4b represents the effect of addition of APE without stress and Fig. 4d the effect of addition of APE in the presence of hydrogen peroxide. The data represent the mean of two independent replicates for each condition (control, 2 mM H_2_O_2_, 0.7 g/L APE, and 0.7 g/L APE + 2 mM H_2_O_2_), and error bars indicate standard deviations from mean

**Fig. 5:**
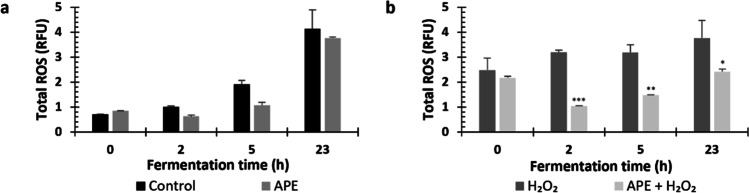
ROS production in yeast during shake-flasks fermentations under hydrogen peroxide (H_2_O_2_) stress with and without antioxidant peptide extract (APE) addition. **a** and **b** Total ROS were presented in relative fold units (RFU) variation during shake-flask fermentation in the absence or presence of 2 mM of H_2_O_2_ and 0.7 g/L APE. The data represents the mean of two independent replicates for each condition, and error bars indicate standard deviations from the mean. Statistically significant differences at each time point were determined using two-way ANOVA, followed by Dunnett’s multiple comparisons test (****p < 0.0001; ***p < 0.001; **p < 0.01; *p < 0.05)

To study the effect of APE in small scale fermentation, shake-flask assays were conducted with 0.7 g/L APE as potential antioxidant and 2 mM H_2_O_2_ as stress inducer. Therefore, shake-flask fermentations were run using four different mediums, standard medium (control), medium with 0.7 g/L APE, medium with 2 mM H_2_O_2_, and medium with 0.7 g/L APE plus 2 mM H_2_O_2_. Results on yeast growth, farnesene concentration, membrane integrity, and intracellular ROS obtained during shake-flask fermentations are presented in Figs. 4 and 5.

In flasks without stress, similar variation on wOD, farnesene concentration, and sucrose consumption were observed between control and APE conditions (Fig. 4a and b). However, in flasks with stress, addition of 0.7 g/L APE led to a significant improvement on yeast growth and farnesene production (Fig. 4c and d). Cell density during exponential phase in shake-flasks with 0.7 g/L of APE plus 2 mM H_2_O_2_ was almost double than in flasks with only 2 mM H_2_O_2_ (Fig. 4c). Sucrose consumption followed the same tendency, being higher in flasks with APE. In addition, farnesene concentration before sugar depletion, at 30.5 h, was 2.8-fold higher in shake-flasks with 0.7 g/L APE and 2 mM H_2_O_2_ than in shake-flasks with only 2 mM H_2_O_2_ (79.4% vs 28.5% of the maximum value in control condition) (Fig. 4d).

Membrane integrity was significantly improved by APE addition in the presence and absence of stress (Table 1). This improvement in shake-flaks under stress was higher at 5 and 23 h. Regarding oxidative stress, the reduction in ROS levels observed in the condition 0.7 g/L APE, in comparison to control, was not statistically significant (Fig. 5a). However, in the presence of 2 mM H_2_O_2_ (Fig. 5b), ROS levels were 1.2- to 3-fold significantly lower in shake-flask with 0.7 g/L APE addition than in shake-flask without antioxidants addition, during exponential phase of fermentation, respectively. The elevated standard deviation in the control at 23 h can be due to small variations in media volume or aeration between shake-flasks replicates (Fig. 5). In sum, the supplementation of *S. cerevisiae* fermentation with 0.7 g/L APE could improve yeast tolerance to oxidative stress and promote yeast growth, under demanding stress environment.

**Table 1:** Membrane integrity (%) in yeast during shake-flask fermentations in standard medium (control), in medium containing antioxidant peptide extract (APE), and under hydrogen peroxide (H_2_O_2_) stress with and without APE addition in the absence or presence of 2 mM of H_2_O_2_ and 0.7 g/L APE

Fermentation time (h)	Control	0.7 g/L APE	2 mM H_2_O_2_	0.7 g/L APE + H_2_O_2_
0	96.64% ± 0.001	96.53% ± 0.001	96.57% ± 0.002	96.38% ± 0.000
2	95.75% ± 0.001	96.19% ± 0.000 ^*^	95.21% ± 0.000	95.37% ± 0.002
5	96.44% ± 0.000	96.84% ± 0.001 ^*^	94.23% ± 0.001	95.38% ± 0.001 ^****^
23	98.41% ± 0.001	99.18% ± 0.000 ^****^	96.55% ± 0.000	99.05% ± 0.000 ^****^

The data represents the mean of two independent replicates for each condition and respective standard deviations. APE results were compared to control results, while APE + H_2_O_2_ results were compared to H_2_O_2_ data. Statistically significant differences at each time point were determined using two-way ANOVA, followed by Dunnett’s multiple comparisons test (****p < 0.0001; *p < 0.05)

### APE protects yeast from stress in bioreactor fermentation

Intracellular ROS generation in yeast can change with several conditions, namely, cellular aging, oxygen transfer rate, and ethanol concentration. Thus, in order to confirm if APE protective effect to yeast stress extends to larger fermentation scales, batch fermentations on 2.7 L bioreactors were performed.

**Fig. 6:**
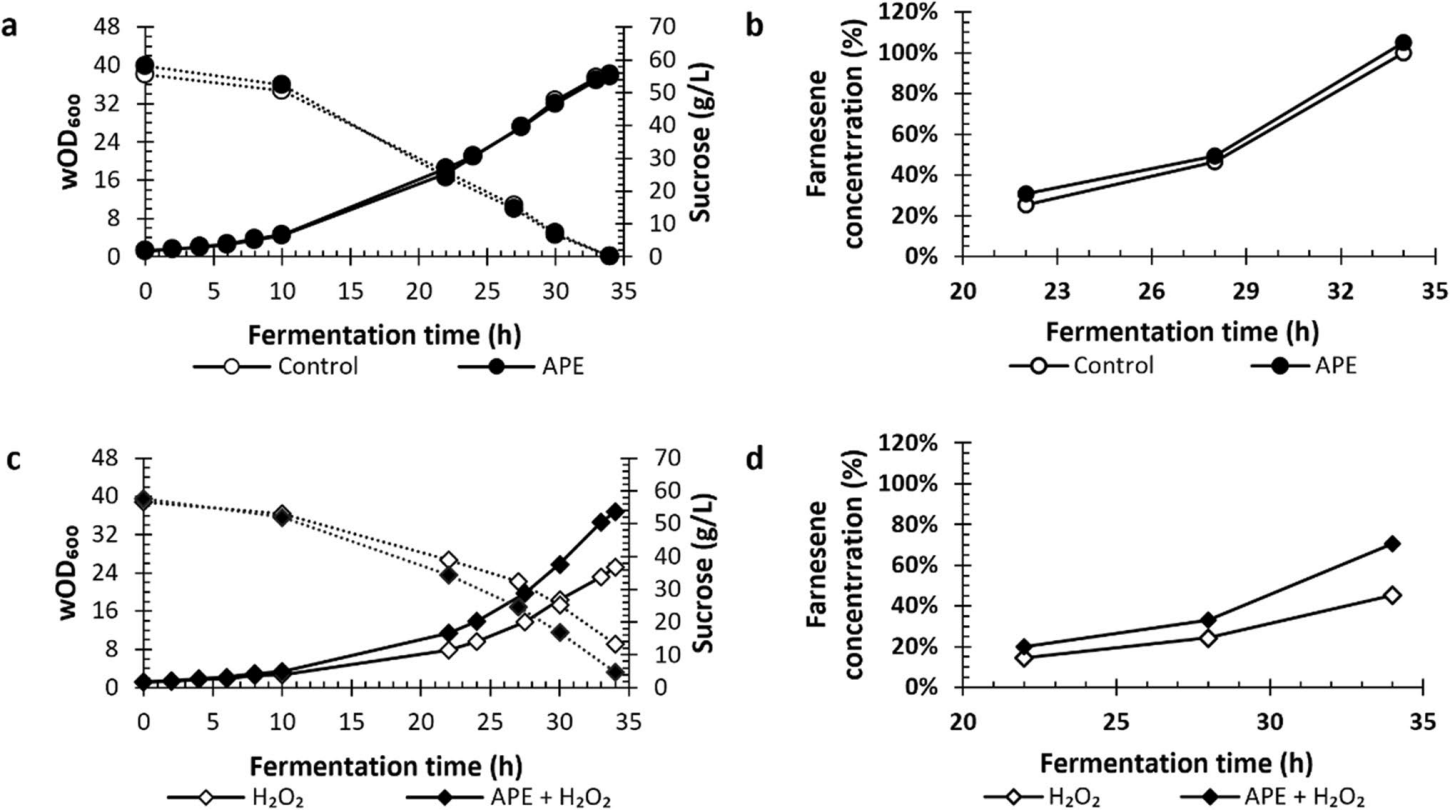
Growth curve (washed optical density at 600 nm — wOD_600_) and sucrose (g/L), over fermentation time for the conditions: control (no extract, no hydrogen peroxide (H_2_O_2_) added), antioxidant peptide extract (APE) at 0.7 g/L, 2 mM H_2_O_2_, and 2 mM H_2_O_2_ plus APE at 0.7 g/L (**a** and **c**). Farnesene concentration (%) relative to control at 34 h of fermentation for the conditions: control (no extract, no H_2_O_2_ added), APE extract at 0.7 g/L, 2 mM H_2_O_2_, and 2 mM H_2_O_2_ plus APE at 0.7 g/L (**b** and **d**). The data represents the mean of two measurements for each condition, and error bars indicate standard deviations from the mean

**Fig. 7:**
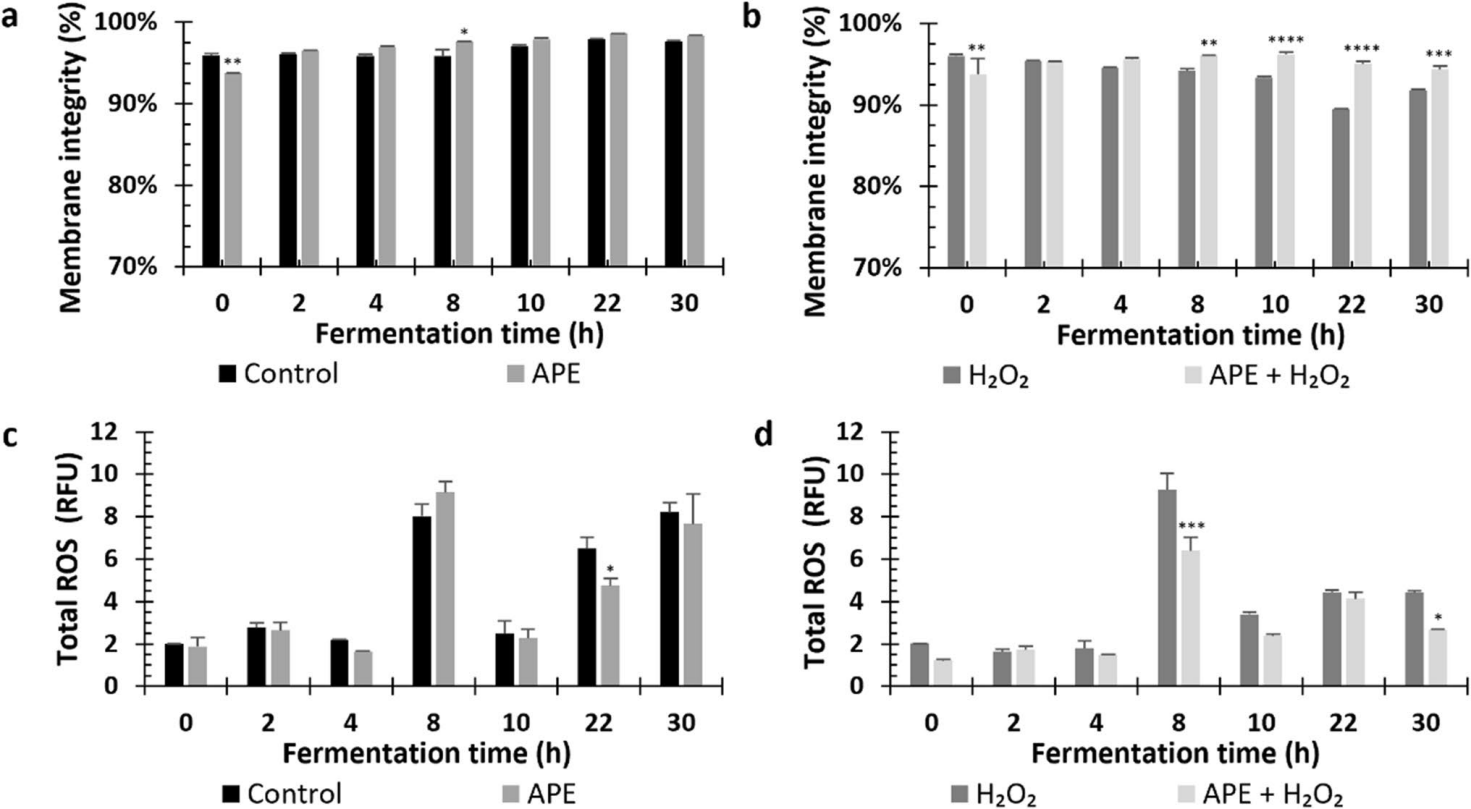
Membrane integrity and total ROS production in bioreactor fermentation under hydrogen peroxide (H_2_O_2_) stress and antioxidant peptide extract (APE) presence and absence. Membrane integrity in each condition tested (**a** and **b**). Variations on total ROS in conditions without stress addition (control and 0.7 g/L APE) (**c**) and conditions with stress (2 mM H_2_O_2_ and 0.7 g/L APE plus 2 mM H_2_O_2_) (**d**) over fermentation time. RFU represents the relative fold units determined in comparation with the cells analyzed in flow cytometer without the dihydrorhodamine 123 probe. The data represents the mean of two measurements for each condition, and error bars indicate standard deviations from the mean. Statistically significant differences at each time point were determined using two-way ANOVA, followed by Dunnett’s multiple comparisons test (****p < 0.0001; ***p < 0.001; **p < 0.01; *p < 0.05)

As in shake-flask experiments, in batch bioreactor fermentations, the addition of 0.7 g/L APE into the culture medium alleviated the negative effect of the stress inducer H_2_O_2_. Relevant positive effects were observed on yeast growth, sugar consumption, and farnesene concentration (Figs. 6 and 7).

Bioreactors without stress factor (control vs 0.7 g/L APE) showed similar growth, sucrose consumption, and farnesene production during the entire fermentation (Fig. 6a and b). Nevertheless, in bioreactors under 2 mM H_2_O_2_, during the exponential phase (6 to 34 h), cell density was up to 1.5-fold higher in the presence of 0.7 g/L APE than in the absence of APE and sucrose consumption was faster (Fig. 6c). At 34 h of fermentation, bioreactor with 0.7 g/L APE plus 2 mM H_2_O_2_ achieved wOD_600_ of 36.75, consumed 52.81 g/L of sucrose, and had farnesene concentration of 70% of that in control condition (i.e., no extract, no stress induced), while bioreactor with 2 mM H_2_O_2_ only reached a wOD_600_ of 25.25, consumed 43.22 g/L of sucrose, and achieved farnesene concentration of 45% (Fig. 6c and d). Thus, the APE supplement led to an improvement of product formation of up to 1.6-fold when cells were subjected to stress. These observations can be attributed to a reduction in oxidative stress promoted by the APE, since the ROS were kept at lower levels when this natural antioxidant was present (Fig. 7).

In bioreactors without H_2_O_2_, APE addition only improved membrane integrity at 8 h of fermentation (Fig. 7a). However, in bioreactors under stress, membrane integrity was significantly increased by APE during exponential phase of fermentation (Fig. 7b). The range in membrane integrity in H_2_O_2_ condition was 96–89%, whereas in H_2_O_2_ plus APE condition that range was 96–94%. At 22 h of fermentation, membrane integrity was 5.5% higher in APE plus H_2_O_2_ compared to H_2_O_2_ condition.

In absence of stress, ROS levels were similar during most of the fermentation time, with no significant differences obtained, except at 22 h of fermentation where APE lead to a significant decrease in ROS levels (Fig. 7c). Although under stress condition a reduction in ROS levels between 1.4 and 1.7-fold was seen in most of the time points analyzed (Fig. 7d), this effect was only statistically significant at 8 and 30 h of fermentation. The higher difference in ROS values was at 8 h of fermentation from 9.27 RFUs, in 2 mM H_2_O_2_ bioreactor, to 6.40 RFUs in bioreactor with 2 mM H_2_O_2_ and 0.7 g/L APE.

According to the results, APE per se did not interfere with the fermentation, since when it was added to the culture medium, the obtained overall fermentation profile was similar to that on standard fermentation condition (i.e., control) (Figs. 6 and 7). Specifically, the APE, at the concentration of 0.7 g/L, did not promote significant differences on yeast growth, sugar consumption, and farnesene concentration over fermentation time in bioreactor batch fermentation. This confirms that the positive effect of APE seen under induced oxidative stress is based on its antioxidant properties and not on an additive effect.

## Discussion

The biotechnology industry is gaining increasing importance in the production of relevant molecules to fulfill the needs of nowadays society while presenting a sustainable alternative to traditional industry such as petrochemical (Chen et al. 2020; Nandy and Srivastava 2018; Coelho et al. 2019; Stalidzans and Dace 2021). Cutting edge fermentation technology has enable the creation of different solutions, ranging from bacteria to eukaryotes, able to produce molecules of high value for different applications such as pharmaceutics, cosmetics, food and beverages, chemicals, plastics, and fuels (biofuels) (Chen et al. 2020; Mendes et al. 2015; Nandy and Srivastava 2018; Qiu et al. 2019). Given the importance of this technology for the transition to a sustainable and carbon-emission free paradigm, it is crucial to continue its development, optimizing processes and reducing production costs (Qiu et al. 2019; Stalidzans and Dace 2021; Takagi 2021; Walker and Basso 2020). On the other hand, this kind of industry also produces waste streams that should be taken into consideration and can be further valued and utilized in a circular economy approach (Faustino et al. 2021; Oliveira et al. 2022a,c; Pinto et al. 2015; San Martin et al. 2021).

The aim of this work was to study the impact of an antioxidant peptide extract (APE) isolated from industrial spent yeast to protect a farnesene producer yeast against stress in fermentation. Industrial fermentations are very demanding environments as yeasts are exposed to several stress factors which can be of physical, chemical, or biological origin (Deparis et al. 2017; Saini et al. 2018; Takagi 2021; Walker and Basso 2020). These factors have a negative impact in fermentation productivity yields; thus, it is of extreme importance to find solutions to overcome issues related to yeast stress. The APE used in this work was characterized elsewhere (Oliveira et al. 2022b), and given its origin and antioxidant properties, we wonder if it could protect yeast against an external stress, particularly, oxidative stress. The generation of ROS is a natural result of cellular aging, thus leading to cell senescence phenotype which hinder productivity (Eigenfeld et al. 2021).

To study this effect, yeast cells were exposed to different concentrations of hydrogen peroxide (H_2_O_2_ at 2 mM or 5 mM), commonly used as oxidative stress inducer, at different exposure periods (1 h or 5 h), in the presence or absence of APE. As positive control AA was used since it is commonly accepted as a potent antioxidant compound (Eigenfeld et al. 2021). Our results showed that APE was able to promote cell growth in the presence of the stress agent in a similar manner to AA (Fig. 2). This effect was more evident when using 2 mM of H_2_O_2_ for both 1 h and 5 h. In these conditions, membrane integrity was kept at acceptable values (> 90%) assuring that cells were still viable (Fig. 3 a and b). The use of 5 mM H_2_O_2_ for 5 h drastically disrupted the yeast growth by decreasing membrane integrity to very low levels (60%, data not shown) which unable the observation of the effects of both APE and AA. The protection against the stress agent by the capacity of APE to decrease the generation of intracellular ROS triggered by the presence of H_2_O_2_ was further evaluated. Following the same tendency of the positive control, APE lost its protective effect when cells were incubated with 5 mM H_2_O_2_. Previous studies also reported that sub-lethal H_2_O_2_ concentrations on yeast cells are lower than 0.4 mM, in which cells can adapt and tolerate, while concentrations higher than 1–2 mM can be lethal (Poljak et al. 2003). In a study with a 5-mM H_2_O_2_ exposure, yeast cell growth was drastically inhibited, and cell density decreased, as consequence of programmed cell death (apoptosis) or irreversible cell injury (necrosis) induced by H_2_O_2_ (Khan et al. 2005; Pedreño et al. 2002). Furthermore, upon exposure to 5 mM H_2_O_2_, the accumulation of protein carbonyls, indicators of oxidative proteins, increased (Khan et al. 2005). The results herein presented are in accordance with literature, which show that 5 mM H_2_O_2_ concentration (especially, for 5 h exposure) is strongly fatal for the yeast cells studied. Nevertheless, APE proved to decrease ROS production even at 5 h of exposure with 2 mM H_2_O_2_ indicating its potent antioxidant activity (Fig. 3e).

Afterwards, the potential antioxidant effect of APE on the fermentation performance of farnesene yeast producer in the presence of the oxidative stress factor was evaluated in small scale (shake-flasks) and bioreactor fermentations. In shake-flasks, the addition of 0.7 g/L APE to the culture medium in the presence of the stress factor improved yeast growth and farnesene production (Fig. 4 c and d). Membrane integrity was also improved by APE addition, especially at 5 and 23 h of fermentation (Table 1). Regarding ROS levels, APE promoted a decrease ranging from 1.2- up to threefold, until sugar depletion (Fig. 5b). The observed effects of APE under stress condition can be attributed to its antioxidant activity, as in other studies peptide extracts have shown to influence repair pathways against oxidative stress caused by H_2_O_2_ or UV radiation (Butylina et al. 2007; Mirzaei et al. 2019). Furthermore, the peptide extract used in the present study led to only 0.05 g/L of extra sugars and trace amounts of minerals in the culture medium, which was not considered significant for yeast growth and product titer. Of note, when APE was added to the normal condition, no relevant effects on yeast growth and farnesene production were observed. Moreover, total ROS levels in control condition were not significantly different from that observed in APE condition (Fig. 4a).

In bioreactor fermentation, the presence of APE in the medium acted similarly as in small-scale fermentations by presenting positive effects on yeast growth and sugar consumption while decreasing the levels of ROS generated by the presence of H_2_O_2_ (Figs. 6 and 7). In the presence of the stress factor, it was also observed that APE restored membrane integrity (Fig. 7b). Importantly, farnesene production was improved when medium was supplemented with APE in the presence of H_2_O_2_ (Fig. 6d). During large-scale (bioreactor) fermentation, oxidative stress has been generally associated with high levels of oxygen transfer rate (by means of high rates of aeration and agitation speeds) resulting in the production of ROS inside the cells (Siddiquee et al. 2019). By introducing 2 mM H_2_O_2_ into culture medium, this stress condition was elevated, as corroborated by ROS results in control condition of bioreactor batch experiment, where higher ROS values were reached than in shake-flasks (especially at 8, 22, and 30 h). Although ROS generation was superior in bioreactor experiment, 1.6-fold increment in farnesene production was observed in culture subjected to stress with APE supplement.

Overcoming oxidative stress is important not only to prevent cell aging but also to respond to other stress factors. Burphan and colleagues (2018) have demonstrated the importance of Cu/Zn-superoxide dismutase (SOD1) for yeast tolerance against different stresses occurring during industrial fermentation. The generation of H_2_O_2_ is a response to the oxidative stress caused by endogenous sources such as normal metabolism (Ceriello et al. 2016) but also of exogenous origins such high extracellular sugar concentration (Semchyshyn et al. 2011) or heat stress (Davidson and Schiestl 2001). The extraction and use of antioxidant peptides from different origins for evaluation of yeast protection against oxidative stress have been evaluated (San Martin et al. 2021; Coelho et al. 2019). For example, four of the eight antioxidant peptide fractions of chia (0.5 g/L) tested by Coelho et al. (2019) promoted an increase of yeast survival rate (16–27%) in the presence 0.75 mM H_2_O_2_. However, to best of our knowledge, this is the first report of the use of antioxidant peptides of yeast origin to improve fermentation and yeast performance with industrial relevance. The APE used in this work has an antioxidant potential around 500 µmol 6-hydroxy-2,5,7,8-tetramethylchroman-2-carboxylic acid (Trolox) /mg protein with 2,2′-azinobis(3-ethyl-benzothiazoline-6-sulphonate)-radical (ABTS^+^) scavenging assay but the mechanism of oxidative protection might involve other mechanisms than just ROS direct scavenging, such as inhibition of lipid oxidation (Oliveira et al. 2022b). Xu and colleagues have shown that of the 20 essential amino acids, 7 have antioxidant activity namely tryptophan, methionine, histidine, lysine, cysteine, arginine, and tyrosine (Xu et al. 2017). The APE used has 5 of these 7 amino acids comprising approximately 15% of the total protein content (Oliveira et al. 2022b). Furthermore, it is well known that arginine, a nutritionally important amino acid, is involved in stress resistance in various organisms (Ji et al. 2020; Nishimura et al. 2010). Thus, it might be hypothesized that the APE is not only scavenging ROS by direct action but also may stimulate other yeast stress defense mechanisms.

Overall, the results herein presented demonstrate that the supplementation of farnesene fermentation process with peptides recovered from industrial spent yeast is a promising strategy to reduce ROS generation, and thus, improve yeast tolerance to oxidative stress while increasing biomolecule productivity. Since APE was obtained from Amyris Inc., Emeryville, CA, USA, fermentation waste, its application to reduce yeast oxidative stress in industrial fermentation may allow to increase process profitability, while promoting both, the valorization of spent yeast waste stream, and the improvement of yeast performance and productivity. Also important, from a circular economy perspective, the valorization of waste streams is also a valuable contribution towards sustainable and eco-friendly solutions for the biotechnology industry. To the best knowledge of the authors, this is the first time that this type of peptide extract is used to overcome oxidative stress in farnesene fermentation.

## Data Availability

Not applicable.
